# Measures of Cultural Competence in Nurses: An Integrative Review

**DOI:** 10.1155/2013/289101

**Published:** 2013-05-30

**Authors:** Collette Loftin, Vicki Hartin, Marietta Branson, Helen Reyes

**Affiliations:** Department of Nursing, West Texas A&M University, Canyon, TX 79016, USA

## Abstract

*Background.* There is limited literature available identifying and describing the instruments that measure cultural competence in nursing students and nursing professionals. *Design.* An integrative review was undertaken to identify the characteristics common to these instruments, examine their psychometric properties, and identify the concepts these instruments are designed to measure. *Method.* There were eleven instruments identified that measure cultural competence in nursing. Of these eleven instruments, four had been thoroughly tested in either initial development or in subsequent testing, with developers providing extensive details of the testing. *Results.* The current literature identifies that the instruments to assess cultural competence in nurses and nursing students are self-administered and based on individuals' perceptions. The instruments are commonly utilized to test the effectiveness of educational programs designed to increase cultural competence. *Conclusions.* The reviewed instruments measure nurses' self-perceptions or self-reported level of cultural competence but offer no objective measure of culturally competent care from a patient's perspective which can be problematic. Comparison of instruments reveals that they are based on a variety of conceptual frameworks and that multiple factors should be considered when deciding which instrument to use.

## 1. Introduction

The United States (USA) is rapidly becoming one of the most racially and ethnically diverse nations in the world. Should this trend continue, minorities are projected to comprise 57 percent of the USA population by 2060. As these numbers continue to grow, achieving greater cultural competence among health care professionals in an effort to meet the health care needs of this diverse population becomes an increasingly critical goal.

*Background.* Registered nurses represent the largest number of  healthcare professionals. However, the racial and ethnic diversity of the current nursing workforce is not reflective of the general population. Findings from the 2008 Sample Survey of Registered Nurses show that although minorities currently constitute 37 percent of the nation's population, minority nurses make up only 16.8 percent of the total nurse population [1].

It has long been recognized that those from racially and ethnically diverse populations suffer higher rates of illness and disability and have experienced reduced access to health care when compared to the overall population [2, 3]. While some progress has been made in reducing health disparities, ongoing problems exist and challenge the USA health care system. Examples of these disparities include higher infant mortality rates for babies born to black women, higher incidence of coronary heart disease and stroke, diabetes, asthma and cancer among minority populations, and increased rates of new human immunodeficiency virus diagnoses among racial and ethnic minorities [3]. Though the causes of these disparities are multifaceted, improvements in cultural competence education for nurses and other healthcare providers are considered to be among the most critical and potentially effective interventions needed to reverse these circumstances [4]. 

The need for culturally competent health care has become an international concern given the recent and escalating growth in global migration. According to Jeffreys [5], the need for culturally competent care has been reported in the international nursing literature. Although this review addresses instruments designed to measure cultural competence in the USA nursing literature, this need has been recognized in the literature from Australia, Canada, Israel, Sweden, South Africa, Great Britain, and others [5]. Several of the reviewed instruments have been translated into multiple languages, including Hebrew, Japanese, Finnish, Swedish, and German [6].

In light of the changing demographics of the USA, it is imperative that nurses appreciate the impact of culture on health. Individuals' culture and ethnicity impart values and beliefs that form the basis for much of their behavior, emotion, and lifestyle. Because clients possess these beliefs and customs based on cultural norms that encompass the many facets of health and illness, it is essential that all health care providers be able to provide care that acknowledges this influence [7, 8]

Cultural competence has been defined in a variety of ways but usually is understood as one possessing the attitudes, knowledge, and skill necessary for providing quality care to a diverse population; on other words, the capacity to deliver culturally appropriate care [9]. In an effort to promote culturally competent health care, nursing leaders have developed a clearly articulated set of standards necessary for providing culturally appropriate nursing care. The twelve standards have been designed to serve as a guide for nurses by emphasizing culturally competent care as a priority for all patients [10]. 

Caring for racially and ethnically diverse populations requires the need for cultural competence training and educational programs. A variety of models describing cultural competence's multiple dimensions and instruments to measure it have become a focus of attention over the past several decades. In 2008, the American Association of Colleges of Nursing (AACN) set out to reinforce the cultural competency elements of  *The Essentials of  Baccalaureate Nursing Education*. USA nursing programs have added or increased the cultural content in their curriculum as a result of this attention and the increasing certainty that the cultural competence of nurses is central to ensuring quality care to all people [11]. In addition, continuing education programs have also highlighted cultural competence training for the existing nursing workforce [12, 13]. 

*Conceptual Definition of Cultural Competence.* Cultural competence has been defined in the literature by multiple disciplines and organizations [39]. For this review, cultural competence is defined as follows:

 
*having the knowledge, understanding, and skills about a diverse cultural group that allows the health care provider to provide acceptable cultural care. Competence is an ongoing process that involves accepting and respecting differences [40]. *

*Research Questions.* Utilizing models of cultural competence, self-efficacy, and the relevant nursing literature, researchers have defined conceptual domains of cultural competence including awareness, knowledge, sensitivity, attitudes, desire, and skills [8, 41]. From these definitions, a variety of instruments to assess the cultural competence of health care providers have been developed. In this review, the instruments were identified and assessed using the following three questions.

 (1) What are the common characteristics of instruments that have been used to measure cultural competence in nurses and nursing students including stated purpose, conceptual framework, and methodology?
 (2) What are the published psychometric properties of the identified instruments utilized to measure the cultural competence of nurses and nursing students?
 (3) What are the concepts of cultural competence the instrument intends to measure?

*Design and Search Methods.* There has been much published in peer-reviewed journals regarding the cultural competence of health care providers. This review is limited, however, to the literature centered on the measurement of cultural competence in nurses and nursing students. A comprehensive search of the literature was performed to locate articles about measurement of cultural competence designed specifically for nursing, adapted for nursing, or suggested for nursing. The following online databases were utilized in this search: Cumulative Index of Nursing and Allied Health Literature (CINHAL), Educational Resources Information Center (ERIC), EBSCO, and FirstSearch and PubMed. The following search terms were used alone and in combination: *cultural competence, cultural competency, cultural instruments, measurement of culture competency, nursing, nursing students,* and cultural *sensitivity*. Once an instrument was identified, it was added as an additional search term. Exclusion criterion included the literature from disciplines outside of nursing that did not specify nurses or nursing students as among the intended users for the instrument. Additional articles were identified through reference lists. The search yielded 427 articles and 41 instruments. The articles were scanned for appropriate terminology to indicate a possible match with the subject under study. The majority of these articles were excluded because they made no mention of nursing or nurses, instead referring to medical students, physicians, managed care organizations, allied health professionals, mental health care providers, and others. Finally, 32 articles testing 11 instruments were considered appropriate and included in this review. 

*Limitations.* This integrative review does have some limitations. Despite a thorough review of the literature for cultural competency instruments for use by nursing researchers and educators, this search may not have identified all available cultural competency scales and subsequent use in testing. Additionally, all subsequent testing of identified scales may not have been located for inclusion in this review. Additionally, cultural competency instrument searches were limited to English language studies.

## 2. Instruments to Measure Cultural Competence

Eleven instruments were identified that assess cultural competence in nurses and nursing students (see Table 1). The following sections provide a description of each instrument's characteristics, psychometric properties and included concepts related to cultural competence. 

One of the earliest instruments, the Cultural Self-Efficacy Scale (CSES), was created by Bernal and Froman [14]. First developed to measure the confidence level of nurses providing care for three ethnic groups—Puerto Rican, African American, and Asian Pacific Islanders—the CSES was later revised to assess confidence in caring for Hispanic, Native American, and Middle Eastern individuals. The CSES items are grouped into three categories: knowledge of cultural concepts, comfort in performing cultural nursing skills, and knowledge of cultural patterns for specific ethnic groups [42]. The cultural specificity of this instrument has been found to limit its use for assessment of nurses caring for individuals from cultures other than those addressed by the instrument [12]. The framework utilized for this instrument is social cognitive theory, specifically the concept of self efficacy [14]. Although it is not linked to an overarching cultural competence model, according to Capell et al. [12], the CSES has been found to be congruent with Giger and Davidhizer's Transcultural Assessment Model and Theory. The 30-item CSES utilizes a Likert-type scale, rating answers from 1 (very little confidence) to 5 (quite a lot of confidence). The authors reported a Cronbach's alpha of .97, and content validity was verified by an expert panel of public health nurses [14]. Factor analysis revealed that four meaningful factors accounted for 90% of the total item covariance: cultural skill, Black cultural self efficacy, Latino cultural self efficacy, and Asian cultural self-efficacy [14]. Coffman et al. [16] found 26 subsequent uses of the CSES. Eight of these were published in peer-reviewed journals, and six of the authors published reliability coefficients ranging from .86 to .98. The tool has been most widely tested among hospital nurses, community health nurses, and nursing students [16]. Similar results reported by Quine et al. [17] identified alpha coefficients of .95 for the knowledge category and .87 for the skills category. The tool is available on the Oncology Nursing Society (ONS) website [43], and it is one of the most frequently used instruments for measuring cultural competence [44]. 

The Transcultural Self-Efficacy Tool (TSET) was developed and tested by Jeffreys and Smodlaka [18, 45] and Jeffreys [19]. This instrument consists of 83 items subdivided into 3 sections: cognitive, practical, and affective. The cognitive subscale rates the respondents' self efficacy in regard to their knowledge of factors influencing nursing care of diverse individuals. The practical subscale measures the respondents' self efficacy in interviewing diverse individuals and includes items such as language, religion, and attitudes about health and illness. Finally, the third subscale rates the respondent's self efficacy in regard to their cultural awareness, acceptance, and appreciation [19, 21]. The TSET utilizes a Likert-type scale, rating answers from 1 (not confident) to 10 (totally confident). part of an overall package of cultural competence development training, this instrument was designed as a diagnostic tool to measure and evaluate nursing students' perceptions of self-efficacy concerning cultural care of patients from diverse backgrounds [19]. According to the authors, the TSET is conceptually based on Bandura's Social Learning Theory and self efficacy as well as a review of the relevant transcultural nursing literature [18]. It corresponds to the model's educational strategy for teaching cultural competence. The model was designed as a method for nurse educators to teach cultural competence to nursing students in an academic setting. Jeffreys and Smodlaka [18, 45] conducted four studies to test the reliability and validity of the TSET. The authors have reported sound reliability and content and construct validity based on their pilot study and five later studies [19, 22, 24]. Reliability testing yielded Cronbach's alphas ranging from .92 to .98, and split halves reliability resulted in coefficients ranging from .76 to .92 [19]. A factor analysis approach analyzed data gathered from 1,260 culturally diverse nursing students, and the results showed that the 83 items were correlated between .30 and .70 [45]. This suggests that all items contributed satisfactorily to the measurement of the construct of transcultural self efficacy. 

A search of CINAHL and PubMed shows that the TSET has been utilized in several dissertations and a single published research article. Lim et al. [21] utilized the instrument in their subsequent study of 196 nursing students. The TSET is currently published in the Jeffrey's 2006 version of *Teaching Cultural Competence in Nursing and Health Care. *In later testing, Jeffreys and Dogan [23] utilized a common exploratory factor analysis with 272 culturally diverse undergraduate nursing students using 69 of the original 83 items. This revealed internal consistency ranging from .94 to .98, and reliability of the instrument was .99. This further suggests that the TSET assesses the multidimensional aspects of transcultural self-efficacy. 

Campinha-Bacote's Inventory for Assessing the Process of Cultural Competence among Healthcare Professional Revised (IAPCC-R) consists of 25 items designed to measure the cultural competence of health care providers in the domains of cultural awareness, cultural desire, cultural knowledge, cultural skill, and cultural encounters. The instrument is based on Campinha-Bacote's Culturally Competent Model of Care [8]. The IAPCC-R is usually completed in 10–15 minutes with scores ranging from 25 to 100. Utilizing a Likert-type scale, the responses range from 1 (strongly disagree) to 4 (strongly agree). Scores indicate whether the healthcare professional is operating at a level of cultural proficiency, cultural competence, cultural awareness, or cultural incompetence. Content validity of the IAPCC-R was established by a panel of experts in culturally competent health care [8]. Construct validity was established using the known-groups technique with a group of 200 registered nurses attending a cultural competence workshop [6]. Reliability has been established by multiple studies [6, 46, 47]. The instrument's author calculates the average reliability coefficient Cronbach's alpha as .83 [44]. The IAPCC-R has been widely utilized in health care research on an international basis. The author maintains a website listing of known uses of the instrument and lists the reliability and validity if reported [6]. There is a list of 20+ published studies on the website, and of these, many report measurement of cultural competence in nurses or nursing students. The remaining measured cultural competence among pharmacists, medical students, optometrists, and allied health professionals. One criticism of the tool is its advanced reading level (e.g., “ethnic pharmacology” and “physiological variations”) making it difficult to utilize when testing disparate educational levels [29]. Although utilized in many health care disciplines, it was designed for testing cultural competence in professional nurses and requires specific knowledge common to the discipline [28]. 

Originally designed for measurement of perceived cultural competence skills in mental health workers, the Ethnic Competency Skills Assessment Instrument (ECSAI) is a 23-item self-report instrument [25]. Napholz modified the ECSAI for use in junior level nursing students. The questionnaire utilizes a Likert-type rating scale with 5 response options ranging from never to always, with a higher score indicating greater cultural competence. Validity was not discussed for this instrument. Reliability was established by a coefficient alpha of .94 [25]. There was no discussion of overarching conceptual framework or of specific concept areas measured by the questionnaire. No subsequent uses of the instrument were identified at the time of this review.

The Cultural Awareness Scale (CAS) developed by Rew et al. [26] was designed to measure cultural awareness in nursing students. The authors considered cultural awareness to be the minimal level of cultural competence. Based on the Pathways Model and found consistent with the Purnell Model of Cultural Competence, the instrument is composed of 36 items [26]. This instrument utilizes a Likert-type scale ranging from 1 (strongly disagree) to 7 (strongly agree). The CAS consists of five subscales: general education experience, cognitive awareness of attitudes, classroom and clinical instruction, research issues, and clinical practice. The internal consistency estimate of reliability was .91 for students and .82 for faculty. Cronbach's alpha for each of the 5 subscales ranged from .66 to .91 for the students and from .56 to .87 for the faculty [26]. A content validity index of .88 was calculated based on data collected from a seven member expert panel. Further validity and reliability testing of the instrument was completed by Krainovich-Miller et al. [27]. Their results were consistent with the findings of Rew et al. [26].

The Cultural Competence Assessment (CCA) was designed to assess the cultural competence of health care providers, including nurses [28]. Based on the Cultural Competence Model of Schim and Miller, the instrument tests the domains of cultural diversity, cultural awareness, cultural sensitivity, and cultural competence behaviors [28]. The CCA is a 26-item instrument utilizing a 5-point Likert-type scale ranging from strongly agree to strongly disagree and no opinion [29]. Tested with hospice nurses, the psychometric properties were sound and support the CCA as an accurate instrument to measure provider cultural competence. The internal consistency reliability of CCA was .89 overall with the two subscales measuring .91 and .75. Construct validity was supported by a factor analysis demonstrating 25 items with loading over .42. 

The Cultural Knowledge Scale (CKS) was designed to evaluate the effectiveness of a cultural competence education program provided for public health nurses [31]. The instrument was designed with items selected from two other previously developed instruments: the IAPCC-R and the CSES [8, 14]. The 24-item CKS utilized a 5-point Likert-type rating scale with response ranging from 1 (strongly agree) to 5 (strongly disagree). The instrument has four subscales: health seeking behaviors, perceptions of health and illness, response to health and illness, and treatment of illness conditions. According to the authors, the Campinha-Bacote [8] Model of Cultural Competence guided the design of the educational intervention and the blending of the items for the instrument. The CKS was considered valid and reliable by the authors because the instrument was generated from items taken from two other instruments with reported validity and reliability. The CKS had a Cronbach's alpha of .71 to .96. 

The Cultural Diversity Questionnaire for Nurse Educators (CDQNE) was developed to measure the cultural competence of nurse educators [32]. The instrument has six subscales: cultural awareness, cultural knowledge, cultural skill, cultural encounters, and cultural teaching behaviors. Based on Campinha-Bacote's Model of Cultural Competence, items for the instrument were written by the authors as well as adapted from other instruments. The 72-item CDQNE utilized a 5-point Likert-type rating scale with responses ranging from 1 (strongly agree) to 5 (strongly disagree). Content validity was determined by a panel of experts. Utilized with a large sample in 2012, no further psychometric properties were reported [33]. 

The Cultural Competency Instrument (CCI) was designed to assess cultural knowledge and competence of clinical researchers, including nurse researchers [35]. It was developed to address the need for culturally competent researchers to participate in a program investigating health disparities in minority populations. It is unique in that this instrument was designed in an effort to produce the specific outcome of increasing African American participation in research projects. The investigators identified an increasing reluctance on the part of African Americans to participate in ongoing research studies [35]. This instrument consists of 20 multiple choice items. There was no conceptual framework identified. No psychometric testing was reported. 

The Cross-Cultural Evaluation Tool (CCET) was used to measure the cultural sensitivity of nursing students before and after the Giger-Davidhizar Model of Transcultural Assessment was introduced during a second-level nursing course [36]. The CCET is a 20-item instrument assessing attitudes and behaviors with a Likert-type rating scale ranging from exhibited always to never demonstrated. A cross-cultural interaction score is obtained, and the score indicates how well the nursing students are able to make culturally sensitive choices. A higher score indicates increased cultural sensitivity. According to Hughes and Hood [36], this instrument was designed by Freeman [37] but not published. Pretest Cronbach's alpha ranged from .73 to .84 across the nursing classes. Cronbach's alpha increases were measured on the posttest scoring from .74 to .87. The instrument was subjected to factor analysis by PCA. Four factors were found to account for 51.9% of variance for the concept cross-cultural interaction. 

The Nurse Cultural Competence Scale (NCCS) was developed by Perng and Watson [38] and is reported by the authors to be based on the literature of Campinha-Bacote, Jeffreys, and others. The scale includes the four domains of cultural awareness, cultural knowledge, cultural sensitivity, and cultural skill. The 41-item NCCS utilizes a 5-point Likert-type scale, with responses ranging from strongly agree to strongly disagree. The authors reported reliability ranging from .78 to .96 for the four subscales during pilot testing. Face validity was established through the review of the scale by nursing experts. A Mokken scaling procedure was performed resulting in a 20-item scale described as reliable by the authors. The final Mokken scale (Cultural Capacity Scale) was comprised of six items from the knowledge scale, two from the sensitivity scale, and twelve from the skill scale. None of the items from the awareness scale were utilized in the final Mokken scale. 

## 3. Discussion

A number of similarities were noted among the instruments. First, the majority of instruments were “culture general,” meaning that there is no distinction made among culture groups [12]. The instruments were developed to assess the health care provider's ability to care for individuals from all diverse backgrounds. However, two of the instruments were “culture specific” [14, 34]. These instruments were developed to assess the ability to care for the needs of individuals from one or more specific ethnic or racial backgrounds. The Cultural Competency Instrument was designed to assess competence of researchers to interact with an African American population, and the Cultural Self-Efficacy Scale was developed to assess confidence levels of nurses caring for Hispanic, African American, and Asian individuals [14]. By classifying assessments in this manner, researchers are able to distinguish the appropriateness of the instrument for specific projects. 

 Each of the instruments was self-administered and measured the self rated cultural competence or some concept of cultural competence attributed to the nurse or nursing student. One of the most significant limitations of the reviewed cultural competence instruments is that they measured the individual's self-perception of cultural competence or cultural self efficacy. The possibility exists that individuals will report what they believe to be the most socially acceptable but perhaps not the most accurate answer [12, 13].

Ten of the eleven reviewed instruments utilized a Likert-type scale. The response options ranged from 4 to 10. Many of the reviewed instruments were utilized to test the effectiveness of an educational or training program in which concepts of cultural competence and the care of diverse individuals were presented. The CCI was utilized to assess the need for such a training program for researchers working with African American adults [34]. The IAPCC-R has also been used in this manner, although it was not designed specifically for evaluating the effectiveness of training [8]. 

Most of the authors reported some level of psychometric testing for the reviewed instruments. Studies on two of the instruments, the CDQNE and CCI, reported no reliability measurements [8, 34]. Measures of internal consistency were reported as .90 or above for the CSES, TSET, ECSAI, CAS, and CCA. The intercorrelations for their reported subscales were more variable, possibly a consequence of the different dimensions of the construct being measured. Five of the instruments had been thoroughly tested in either initial development or in subsequent testing with the developers providing extensive testing details. These instruments were the CSES, TSET, IAPCC-R, CCA, and the NCCS. The psychometric properties of the remaining instruments have not been as extensively evaluated. 

The domains of cultural competence as defined for these instruments vary, although there is overlap. Eight of the eleven instruments assessed *in some measure* the individual health care providers' confidence in or perception of their own skill to care for an individual from a diverse population. Eight of the instruments assessed the caregiver's perception of cultural awareness. All of the instruments measured cultural knowledge. This awareness-knowledge-skill model of cultural competence assessment is common in many disciplines [48]. 

Four of the instruments were based on Campinha-Bacote's [8] model of culturally competent care: the IAPCC-R, CKS, CDQNE, and the NCCS. This model focuses on the provider attributes of cultural awareness, cultural desire, cultural knowledge, cultural skill, and cultural encounters, providing a comprehensive set of concepts to base the instruments [48]. The CSES, TSET, CAS, and the CCA are also based on comprehensive models of cultural competence but with fewer domains. 

For health care providers and specifically nurses, the need to provide culturally appropriate and competent care is recognized as essential in light of the growing diversity among individuals they care for. Still, great difficulty exists in assessing the cultural competence of providers. Currently, the instruments to assess cultural competence in nurses and nursing students are self-administered and based on individuals' own perceptions. 

Development of awareness and skill can be acquired through education and training, which Jeffreys [5] considers an integral component in the development of cultural competence. However, development of cultural competence is not immediate; rather it is gradual. Cultural competence is an ongoing process [8]. As a way of assessing cultural competence, Campinha-Bacote [8] recommends ongoing training and staff development with multiple assessments over a period of time. Much of the testing of these instruments has relied heavily on convenience samples of nurses or nursing students who were readily available. This sampling technique is a major limitation on ability to generalize the results to other groups of nurses or students.

The AACN has described three characteristics of culturally competent baccalaureates [11]. These characteristics are awareness of personal culture, values, beliefs, attitudes, and behaviors; skill in assessing and communicating with individuals from other cultures; and assessment of cross-cultural variations. The CSES, CAS, CCA, and the CDQNE measure the self-perception and self efficacy of two of the three constructs, awareness and skill, while the TSET and IAPCC-R measure all three. 

These instruments provide a method of assessment and reassessment that is readily available and easily administered. Several of the instruments can be administered in as few as 10–15 minutes [8, 28]. It has been suggested that further development of these assessments includes some objective measures or perspectives of the client in an effort to provide a more complete assessment of the nurse's cultural competence [49]. Without some measure to assess cultural competence in terms of outcomes for the patients, the nurses' ability not only to claim competence, but also actually to provide culturally competent care will remain unknown. 

Measuring the cultural competence of nurses and nursing students is complex but is becoming an increasingly important aspect of assessing quality care for individuals from diverse groups considering the changing USA demographics. For this reason, the challenges in measuring cultural competence must be addressed. These challenges in evaluating cultural competence in nursing practice and education have led to the development of instruments that focus on the cultural competence attributes of health care providers rather than on patient perceptions of their care or their health outcomes [11, 12]. 

Several of the reviewed instruments including the TSET, IAPCC-R, CSES, and the CCA have been used in multiple studies and in a variety of situations and settings, providing some context for future research endeavors. Hopefully, the identification of instruments and their theoretical and psychometric properties will be valuable for those measuring and testing strategies in efforts to increase the cultural competence of nurses and nursing students. 

Despite the limitations associated with existing instruments, there is much value in the initial assessment of cultural competence they provide as well as tracking measurements of cultural competence over time [11]. Because providing culturally competent care is essential in nursing, the measurement of cultural competence and its effect on patient outcomes is central to the discipline. Cultural competence is still, however, a difficult concept to objectively measure and will continue to be so for nurse researchers and nurse educators. The challenge remains to develop measures to assess cultural competence in practice and on patient outcomes.

## Figures and Tables

**Table 1:** Review of Instruments.

Instrument	Purpose	Subjects	Description of instrument	Stated conceptual framework	Score methods	Reported reliability	Reported validity	Subsequent use/testing	Reliability reported in subsequent testing
(1) Cultural Self-Efficacy Scale (CSES), Bernal and Froman, 1993 [14]	Designed to test perceived sense of self efficacy in caring for diverse patients (Black, Hispanic, and Asian)	Piloted with community health nurses	30 items testing knowledge of cultural concepts, comfort in performing cultural nursing skills, and knowledge of cultural patterns for specific ethnic groups	Consistent with Bandura Self Efficacy (*Social Learning Theory*) and Leininger; otherwise not linked with an overarching conceptual model	Survey—5-point Likert-type scale with 1—very little confidence and 5—quite a lot of confidence	Cronbach's alpha .97 overall with subscales ranging from .85 to .98.	Content validity determined by an expert panel of 5 public health nurses followed by factor analysis confirming construct validity	Jones et al., 2004 [15]	Consistent with pilot study
								Coffman et al., 2004 [16] reported multiple published and unpublished uses of CSES	Reliability reported ranging from .86 to .98
								Quine et al., 2012 [17]	Reliability reported for knowledge subscale .95 and skills subscale .87

(2) Transcultural Self-Efficacy Tool (TSET), Jeffreys and Smodlaka, 1996 [18]	Diagnostic tool designed to evaluate students perceived self efficacy caring for diverse clients	Original pilot study had 357 nursing students	83-item questionnaire with 3 subscales: cognitive, practical, and affective	Bandura—Self Efficacy (*Social Learning Theory*), concepts of transcultural nursing	Survey—10-point Likert-type scale. For all items 1—not confident to 10—totally confident	Total alpha .97 and .98 with subscale alpha ranging from .90 to .98	Content validity by 6 member expert panel followed by factor analysis confirming construct validity	Jeffreys, 2000 [19]	Consistent with pilot study
								Jeffreys and Smodlaka, 1999 [20] Lim et al., 2004 [21]	Published reliability .091–.093 for subscales
								Larsen and Reif, 2011 [22]	Cronbach's alpha ranged from 0.982 to 0.990
								Jeffreys and Dogan, 2010 [23]	CEFA testing internal consistency .94 to .98 and reliability of .99
								Jeffreys and Dogan, 2012 [24]	Authors confirm validity and reliability

(3) Inventory for Assessing the Process of Cultural Competency (IAPCC and IAPCC-R), Campinha-Bacote, 2009 [6]	Designed to measure level of cultural competence in health care providers	Acute care setting with 15 registered nurses	Original scale of 20 items revised scale of 25-item questionnaire based on 5 constructs of desire, awareness, knowledge, skill, and encounters	Based on *The Process of Cultural Competence in the Delivery of Healthcare Services *Campinha-Bacote, 2002 [8]	Survey—4-point Likert-type scale with differing range for responses	Reliability of original version reported as a “limitation”	Content validity determined by 5 national health care experts in fields of transcultural nursing and construct validity determined by known groups technique	Multiple reported uses of IAPCC-R [6]	Consistent with reported psychometric values of revised version The author reports “average” reliability of revised version Cronbach's alpha .83

(4) Ethnic Competency Skills Assessment Inventory— (ECSAI), Napholz, 1999 [25]	Originally designed for another discipline; used to examine self-report cultural competence of nursing students	56 nursing students from 2 campuses	23 items with no description of items	Not reported	Survey—5-point Likert-type scale—never to always	Coefficient alpha .9444	Not reported	Not reported	Not reported

(5) Cultural Awareness Scale (CAS), Rew et al., 2003 [26]	Designed to test cultural awareness of nursing students	Instrument piloted with 72 undergrad and grad nursing students	36-item questionnaire with 5 subscales: general education experience, cognitive awareness, research issues behavior/comfort with interactions, and patient care issues	Based on the *Pathways Model* and consistent with the *Purnell Model of Cultural Competence *	Survey—7-point Likert-type scale—strongly agree to strongly disagree	Cronbach's alpha .82 for faculty and for students .91 on overall test	Content validity determined by 7 member expert panel	Krainovich-Miller et al., 2008 [27]	Overall testing was consistent with original pilot reporting

(6) Cultural Competence Assessment (CCA), Schim et al., 2003 [28].	Designed to measure cultural competence among hospice nurses and workers	Field tested and pilot tested with 51 hospice nurses and caregivers	25 items with subscales—awareness and sensitivity, cultural diverse experiences, and cultural competence behaviors	Based on *Cultural Competence Model* of Schim and Miller	Survey—5-point Likert-type scale—strongly agree to strongly disagree and no opinion	Cronbach's alpha overall was .92 with subscales reliability of .93 and .75	Content and face validity determined by 2 expert panels	Doorenbos et al., 2005 [29]	Tested in health care providers with consistent results
							Construct validity testing by factor analysis with two factors loading over .40 and contrasted group validity was determined	Starr and Wallace, 2009 [30]	Not reported

(7) Cultural Knowledge Scale (CKS) [31]	Designed to evaluate effectiveness of cultural competence training	Convenience sample of 76 public health nurses enrolled in 5-week cultural competence training course	24 items with 2 knowledge subscales—items taken from IAPCC-R and CSES	Based on *The Process of Cultural Competence in the Delivery of Healthcare Services *Campinha-Bacote, 2002 [8]	Survey—5-point Likert-type scale—strongly agree to strongly disagree	Cronbach's alpha of 0.71 to 0.96	Otherwise reliability and validity were based on original instruments from which items were taken	Not reported	Not reported

(8) Cultural Diversity Questionnaire for Nurse Educators— CDQNE [32]	Designed to measure cultural competence of nurse educators	Convenience sample of 163-nursing faculty	72 items with 2 subscale measuring 5 constructs of desire, awareness, knowledge, skill, and encounters—included items from the IAPCC-R	Based on *The Process of Cultural Competence in the Delivery of Healthcare Services *Campinha-Bacote, 2002 [8]	Survey—5-point Likert-type scale—strongly agree to strongly disagree	Not reported	Content validity was determined by a panel of experts	Reneau, 2013 [33]	Not reported

(9) Cultural Competency Instrument (CCI), Kosoko-Lasaki et al., 2006 [34]	Designed to assess provider and investigator knowledge, attitudes, and sensitivity to other cultures	Pilot tested with “small” group of clinical researchers and research nurses	20 items	Not reported	Survey—multiple choice	Not reported	Content validity was based on review of the literature “no attempts” to further validate	O'Brien et al., 2006 [35]	Psychometric values not reported

(10) Cross-Cultural Evaluation Tool (CCET), Hughes and Hood 2007 [36]	Designed to measure the cultural sensitivity of nursing students after educational activity	Convenience sample of 218 nursing students	20 items	Based on items from an unpublished presentation by Freeman, 1993 [37]	Survey—5-point Likert-type rating scale assessing behaviors with range from always exhibited to never demonstrated	Cronbach's alpha of 0.73 to 0.87. Significant alpha increases on posttest	Subjected to factor analysis by PCA. Four factors accounting for 51.9% of variance for the concept cross-cultural interaction	Not reported	Not reported

(11) Nurse Cultural Competence Scale (NCCS), Perng and Watson 2012 [38]	Designed to measure cultural awareness, cultural knowledge, cultural sensitivity, and cultural skill	Pilot testing with 47 Taiwanese on the job nursing students	41 items, measuring 4 constructs: cultural awareness, knowledge, sensitivity, and skill	Based on the literature of Campinha-Bacote, Jeffreys, and others	Survey—5-point Likert-type scale—strongly agree to strongly disagree	Cronbach's alpha for 4 scales ranged from 0.78 to 0.96	Face validity established through reviews by 4 experts	Perng and Watson, 2012 [38]	Not reported
